# Sulfate assimilation in eukaryotes: fusions, relocations and lateral transfers

**DOI:** 10.1186/1471-2148-8-39

**Published:** 2008-02-04

**Authors:** Nicola J Patron, Dion G Durnford, Stanislav Kopriva

**Affiliations:** 1 School of Botany, the University of Melbourne, Victoria 3010, Australia; 2 Department of Biology, University of New Brunswick, Fredericton, New Brunswick, EB3 5A3, Canada; 3 John Innes Centre, Norwich Research Park, Norwich NR4 7UH, UK

## Abstract

**Background:**

The sulfate assimilation pathway is present in photosynthetic organisms, fungi, and many bacteria, providing reduced sulfur for the synthesis of cysteine and methionine and a range of other metabolites. In photosynthetic eukaryotes sulfate is reduced in the plastids whereas in aplastidic eukaryotes the pathway is cytosolic. The only known exception is *Euglena gracilis*, where the pathway is localized in mitochondria. To obtain an insight into the evolution of the sulfate assimilation pathway in eukaryotes and relationships of the differently compartmentalized isoforms we determined the locations of the pathway in lineages for which this was unknown and performed detailed phylogenetic analyses of three enzymes involved in sulfate reduction: ATP sulfurylase (ATPS), adenosine 5'-phosphosulfate reductase (APR) and sulfite reductase (SiR).

**Results:**

The inheritance of ATPS, APR and the related 3'-phosphoadenosine 5'-phosphosulfate reductase (PAPR) are remarkable, with multiple origins in the lineages that comprise the opisthokonts, different isoforms in chlorophytes and streptophytes, gene fusions with other enzymes of the pathway, evidence a eukaryote to prokaryote lateral gene transfer, changes in substrate specificity and two reversals of cellular location of host- and endosymbiont-originating enzymes. We also found that the ATPS and APR active in the mitochondria of *Euglena *were inherited from its secondary, green algal plastid.

**Conclusion:**

Our results reveal a complex history for the enzymes of the sulfate assimilation pathway. Whilst they shed light on the origin of some characterised novelties, such as a recently described novel isoform of APR from Bryophytes and the origin of the pathway active in the mitochondria of Euglenids, the many distinct and novel isoforms identified here represent an excellent resource for detailed biochemical studies of the enzyme structure/function relationships.

## Background

Sulfur is found in all organisms as a constituent of the proteogenic amino acids cysteine and methionine and in many coenzymes and other metabolites. In most of these compounds sulfur is present in a reduced form of organic thiols or sulfides. The major form of sulfur in nature, however, is inorganic sulfate. Sulfate is taken up into cells, reduced, and incorporated into cysteine in the pathway of sulfate assimilation [1,2]. Since sulfate is a stable molecule, for reduction it must be activated. The adenylation to adenosine 5'phosphosulfate (APS) is catalyzed by ATPS. Plant ATPS is a homotetramer of 52–54 kDa polypeptides [3]. Bacterial ATPS, on the other hand, consists of four heterodimers composed from 35 kDa CysD and 53 kDa CysN subunits [4]. Some organisms, such as fungi and many bacteria, require a second activation step, i.e. phosphorylation of APS by APS kinase to 3'phosphoadenosine 5'phosphosulfate (PAPS). In fungi, ATPS and APS kinase are fused into a single 59 to 64 kDa subunit that assemble into a homohexamer [5].

The reduction of activated sulfate consists of two steps. First, APS or PAPS is reduced to sulfite by APR or PAPS reductase (PAPR). APR exists as a homodimer in prokaryotes and it is fused to thioredoxin in plants and green algae [6]. Most APRs contain an FeS cluster, however a variant of the enzyme from early branching streptophyte lineages (bryophytes and lycopodiophytes) has been shown to catalyze APS reduction without the FeS chemistry [6-9]. PAPR is similar to bacterial APR but does not bind the FeS cluster [6,10].

In the second step, sulfite is reduced to sulfide by sulfite reductase (SiR). Plant SiR is a monomeric protein of 65 kDa containing a siroheme and an FeS cluster [11]. In contrast, the bacterial NADPH-SiR is an oligomer of eight 66 kDa flavoprotein subunits (CysJ) and four 64 kDa siroheme and [4Fe-4S] cluster binding hemoproteins (CysI) [12]. SiRs in fungi are composed from α and β subunits of 116 kDa and 167 kDa respectively. Similar to bacterial SiR, the fungal enzyme requires siroheme, FAD and FMN [13]. Sulfide is incorporated into the amino acid skeleton of *O*-acetylserine (OAS) or *O*-acetylhomoserine in fungi, to form cysteine or homocysteine, respectively, in a reaction catalyzed by *O*-acetyl(homo)serine-(thiol)lyase (OASTL). OAS itself is synthesized by acetylation of serine by serine acetyltransferase.

Although sulfate assimilation is essential for the incorporation of reduced sulfur into bioorganic compounds, the pathway seems to be readily dispensable when the lifestyle of the organism allows. It is absent in all metazoans, which satisfy their need for reduced sulfur by the ingestion of proteins to obtain the sulfur containing amino acids cysteine and methionine. Bacterial and protistan parasites also lack a sulfate assimilation pathway. In bacterial species that have undergone a significant genome reduction, the sulfate assimilation operon is almost invariably lost [14,15]. This is enabled by the adaptation of the nutrition of these parasites for metabolites provided by the host. A third group of organisms usually lacking sulfate assimilation are Archaea and bacteria using dissimilatory sulfide (or thiosulfate) oxidation or sulfate reduction for respiration and energy conversion. The habitats of such organisms always contain sulfide [16], therefore, there is no need for sulfate assimilation to sustain cysteine biosynthesis. The enzymes of the dissimilatory pathway, despite catalyzing the same chemical reaction, are either highly divergent (ATPS) or entirely unrelated (APR and SiR) to those of the sulfate assimilation pathway.

In plants, OAS and cysteine are synthesized in all three compartments capable of protein synthesis, i.e. cytosol, plastid, and mitochondria [17]. Sulfate activation, however, takes place in the cytosol and plastids, whereas sulfate reduction is confined to plastids [17,18]. Since sulfite reductase utilizes ferredoxin as an electron donor, the association of sulfate reduction with photosynthesis and plastids might be general for all photosynthetic eukaryotes. However, there is a remarkable exception, the photosynthetic flagellate *Euglena gracilis*, in which activity of enzymes involved in sulfate reduction and cysteine synthesis have been identified in the mitochondria [19-21]. Experiments with purified mitochondria with the outer membrane removed, found that assay products accumulated largely in the medium indicating that in *Euglena *ATPS, APR, and SiR seem to be located in a sulfate metabolizing centre on the outside of the mitochondrial inner membrane [21].

This high variability in the components and locations of sulfate assimilation in eukaryotic lineages provokes the question of the origin of the pathway, especially plastid-containing lineages, which generally reduce sulfate in that organelle. Here we present the results of detailed phylogenetic analyses of each of enzymes that constitute the pathway. In addition we address the sub-cellular localization of the pathway, with a special focus on the secondary-plastid containing genus, *Euglena*.

## Results

### Eukaryotic lineages reduce sulfate in one of three cellular compartments

An investigation of the complete genomes of many eukaryotic species and several others from which extensive expressed sequence tag data has been generated revealed genes for the sulfate assimilation enzymes ATPS, APR/PAPR, and SiR in all eukaryotes with photosynthetic plastids as well as fungi, *Phytopthora *spp. and the choanozoan, *Monosiga brevicollis *(Table 1). In the metazoans, the mycetozoan *Dictyostelium discodium *and the parasitic alveolate *Toxoplasma gondii*, the genes for the reductive part of the pathway are missing, however ATPS and APS kinase are still present. These species do not reduce sulfate but receive cysteine in their nutrition or produce it by transsulfuration of methionine [2]. The sulfate activation enzymes are, however, essential for the synthesis of PAPS, which is the form of activated sulfate required for sulfation reactions. Sulfation of proteins and polysaccharides is a widespread modification. Hence, ATPS and APS kinase are ubiquitous among eukaryotes and in some lineages are fused together into the PAPS synthetase protein [22].

**Table 1:** Numbers of isoforms and cellular locations of ATPS, APSK, APR, PAPR and SiR. Numbers of isoforms and cellular locations, predicted or experimental (bold type) of ATP-sulfurylase (ATPS), APS kinase (APKS), APS reductase (APR), PAPS reductase (PAPR) and sulfite reductase (SiR) found in eukaryotic genomes. CP: plastid; CY; cytosol; MT: mitochondria; § ATPS and APSK proteins are fused; n: activity also detected in nucleus; *: gene model may be truncated at 5' end; a: APR without FeS cofactor binding sites; †: EST survey only; ^x^: Organism does not reduce sulfate; ^p^: fusion to inorganic pyrophosphatase.

	ATPS			APSK		APR			PAPR		SiR		
**NO PLASTID**	CP	CY	MT	CP	CY	CP	CY	MT	CP	CY	CP	CY	MT

METAZOAN													
*H. sapiens*^x^													
MYCETOZOAN		**2§n**			**2§n**								
*D. discoidum *^x^		1			1								
CHOANOFLAGELLATE													
*M. brevicollis*		1§			1§					1		1	
FUNGI													
*S. cerevisiae*		**1**			**1**					1		1	
*A. nidulans*		1			1					**1**		**1**	
HETEROKONT													
*P. sojae*		1§			1§					1		1	

**1° RED PLASTID**	CP	CY	MT	CP	CY	CP	CY	MT	CP	CY	CP	CY	MT

RHODOPHYTE													
*C. merolae*	1	1			1	1					2		

**2° RED PLASTID**	CP	CY	MT	CP	CY	CP	CY	MT	CP	CY	CP	CY	MT

HETEROKONT													
*T. pseudonana*	1§^p^	1		1§	1	2a					1		
HAPTOPHYTE													
*E. huxylei*	1	1§^p^				1a					1		
ALVEOLATE													
*T. gondii*^x^		1			1								

**1° GREEN PLASTID**	CP	CY	MT	CP	CY	CP	CY	MT	CP	CY	CP	CY	MT

CHLOROPHYTE													
*C. reinhardtii*	2			1		1					2		
*O. taurii*	1			1		1					1		
*O. lucimarinus*	1			1		1					1		
BRYOPHYTE													
*P. patens*	2			1	3	**1+1a**					3		
LYCOPODIOPHYTE													
*S. moellendorffii*	1			2	2	**1+1a**					1*		
SPERMOPHYTE													
*A. thaliana*	**4**			2	2	**3**					1		
*O. sativa*	1	1		2	1	2					1		

**2° GREEN PLASTID**	CP	CY	MT	CP	CY	CP	CY	MT	CP	CY	CP	CY	MT

EUGLENIDS													
*E. gracilis*†			**2**					**2**					**1**
CHLORARACHNIOPHYTE													
*B. natans*†	1					1a							

In the species of aplastidic groups, most enzymes lack any N' terminal sub-cellular targeting information and are presumed to be cytosolic. The exceptions are metazoan PAPS synthetases that have been localized both to cytosol and the nucleus [23]. Consistent with biochemical data (reviewed in [1,2]), in species with primary photosynthetic plastids almost all sulfate-reducing enzymes show clear plastid-targeting pre-sequences at the N' terminus as indicated by an N terminal extension (compared to bacterial homologues) and amino acid composition consistent with a plastid transit peptide for each species [24]. The exceptions are isoforms of ATPS and APS kinase from a few plant and red algal species lacking any N' -terminal targeting information and are thus predicted to be cytosolic. These enzymes are probably involved in the production of PAPS for sulfation reactions as in other eukaryotes. Experimental evidence from Arabidopsis and other plant species supports this, with ATPS activity being identified in the plastids and the cytosol but APR activity only found in the plastid [25,26]. Only one of the four isoforms, however, was identified in a proteomic study of the plastid [27]. Anomalously, all four ATPS isoforms identified from Arabidopsis have N' terminal extensions typical of plastid transit peptides.

**Figure 1:**
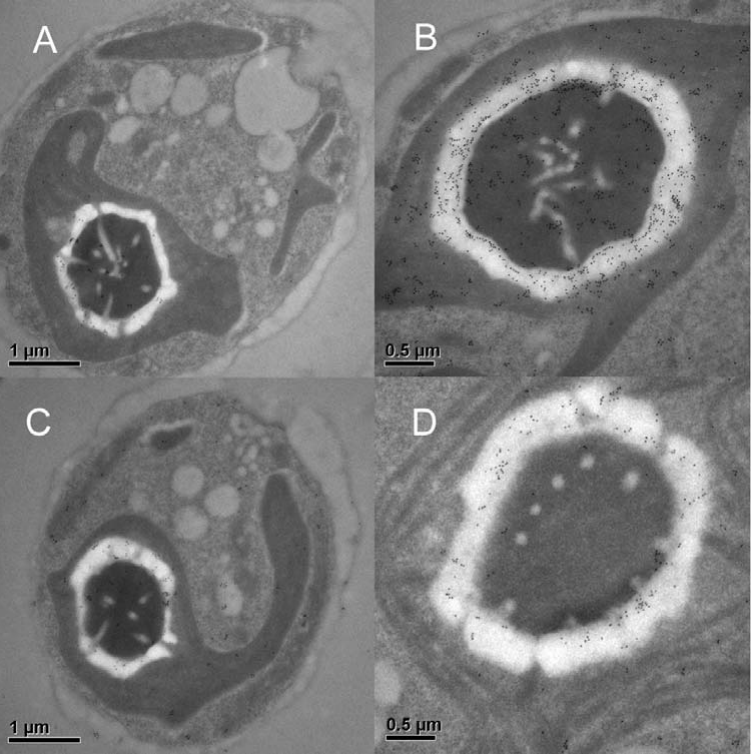
**TEM micrographs of immunogold labeling of *C. reinhardtii *using antisera against APR (1:2500) (A and B) and SiR (1:500) (C and D)**. Panels A and C show whole cells where the gold particles are almost exclusively in the plastid, particularly on the starch sheath surrounding the pyrenoid. Panels B and D show the pyrenoid and surrounding starch sheath, the gold can be seen in an almost continuous ring over the sheath.

In contrast to plants, no biochemical data for the pathway's localization exists for algae. The plastid transit peptides of green algae, especially, are shorter than average [24,28] and prediction software cannot distinguish plastid and mitochondrial targeting pre-sequences [24,28]. Therefore, we addressed the localization of sulfate assimilation in the green alga *Chlamydomonas reinhardtii *by immuno-transmission electron microscopy. In Western blot analysis, antisera against Arabidopsis APR and SiR recognized single protein bands of 50 and 75 kDa, respectively, in *C. reinhardtii *protein extracts (Additional File 1). Signals from both antibodies were found to be almost exclusively within the plastid, indicating that the plastid is the site of sulfate reduction (Figure 1 and Additional File 2). Surprisingly, the signal was not distributed evenly within this compartment and instead was concentrated around the starch sheath, in close association with the pyrenoid (Figure 1). Interestingly, in the bryophyte *P. patens*, localization of APR by immuno-fluorescence of a GFP fusion protein also revealed an uneven distribution of the protein in the plastid [8]. The close co-localization of the two enzymes in *Chlamydomonas *indicates that the enzymes of sulfate activation and reduction may form a multienzyme complex similar to that of serine acetyltransferase and OASTL [17].

**Figure 2:**
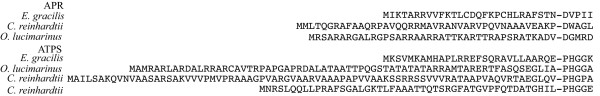
**N terminal regions of ATPS and APR from *E. gracilis *along with their chlorophyte relatives *C. reinhardtii *and *O. lucimarinus*, showing mitochondrial targeting sequences in *Euglena *and plastid targeting sequences in the chlorophytes**. The start of conserved sequence is indicated by a dash.

In algae with secondary plastids, enzymes for sulfate assimilation contained N terminal extensions (signal peptide followed by transit peptide) indicative of location in plastids [29,30] with one exception: enzymes of *E. gracilis *lacked the typical Euglenid tri-partite (signal peptide followed by transit peptide, and stop transfer sequence) N' terminal pre-sequence required for transit to its secondary plastids [31] and instead encoded a pre-sequence typical of mitochondrial targeting (Figure 2). The subcellular localization tools, TargetP and iPSORT, also suggested a mitochondrial location for these proteins. We determined the ATPS transcripts identified from the *E. gracilis *EST project to be full length, as evidenced by the presence of a splice leader. Though the splice leader was not apparent from the APR and SiR ESTs the coding sequences are likely complete due to the presence of an obvious N terminal extension compared to bacterial isoforms. The presence of a mitochondrial pre-sequence is in accordance with biochemical data from that species locating sulfate assimilation to mitochondria [19,21,32].

In order to determine the origin of mitochondria-targeted enzymes found in *Euglena *and to investigate the relationships of the enzymes found in other eukaryotes, particularly to determine if isoforms found in the cytosol of aplastidic eukaryotes are related to those found in plastids, we performed phylogenetic analyses of the enzymes of the pathway.

### Multiple origins and gene-fusions of ATPS

**Figure 3:**
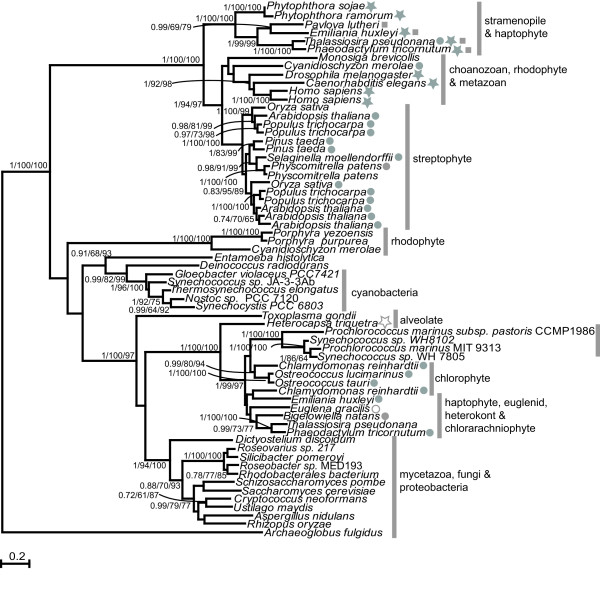
**Maximum likelihood phylogenetic tree of ATPS**. The outgroup is an alignable sequence of a dissimilatory ATPS from archaea. Numbers at nodes are (left to right) Bayesian posterior probabilities, maximum likelihood bootstraps and distance bootstraps. Support for nodes with less that 60 bootstraps by both methods are not show. Where tree topology differed or bootstraps by one method are less than 60, a dash is shown. A filled circle indicates the presence of a plastid targeting presequence; an open circle indicates a mitochondrial targeting presequence; a filled star indicates fusion to APS kinase; an open star indicates fusion to APR; a filled square indicates fusion to inorganic pyrophosphatase. The bar indicates the number of amino acid changes per branch length.

The general distribution of taxa in the ATPS tree is unexpected (Figure 3). Most notably, isoforms encoded by species belonging to well defined groups, such as Opisthokonts (represented in our analysis by fungi, metazoans, mycetazoans and choanozoans), cyanobacteria and Viridiplantae are not monophyletic.

Fungal ATPS shows specific relationship to that found in Rhodobacterales, which unlike other α-proteobacteria lack the bacterial type CysN/CysD enzyme. However, these bacterial ATPSs are not at the base of the clade; that position being occupied by eukaryotic ATPS isoforms (from the slime mold *D. discoidum *and the alveolate *T. gondii) *involved only in the production of PAPS. This, and the fact that no other bacteria are found at the base of the clade but instead in a sisterhood to an ambiguous group containing cyanobacteria and eukaryotes, makes it uncertain if ATPS is of mitochondrial (α-proteobacterial) origin in these eukaryotes or if a lateral gene transfer has taken place at a later date.

Two other Opisthokont lineages, metazoans and the choanozoan *M. brevicolis*, encode an ATPS only distantly related to the fungal one. This isoform is most related to those found in plants and stramenopiles and is fused at the N' terminus to APS kinase. The fusion of ATPS and APS kinase is very common, occurring also in fungi where the APS kinase domain is attached to C terminus of ATPS; however, in fungi the function of the APS kinase domain is regulatory rather than catalytic. The most likely explanation is that ATPS and APS kinase were fused in the ancestral eukaryotes, as supported by the existence of this fused protein in diverse lineages (metazoans, choanozoans, and stramenopiles). The gene must then have split in red algae and plants. If the enzyme found in fungi, *D. discoidum *and *T. gondii *is of α-proteobacterial origin then the ancestor of these groups and those that encode the fusion protein would have contained both isoforms and losses would have occurred multiple times in various lineages.

Unexpectedly, fusions of ATPS with another enzyme were revealed in the stramenopiles *Thalassiosira pseudonana*, *Phaeodactylum tricornutum*, and *Phytophthora sojae *and the haptophytes *Pavlova lutheri *and *Emiliania huxleyi*. In these organisms ATPS is fused to inorganic pyrophosphatase. The hydrolysis of pyrophosphate is an efficient mechanism to shift the very unfavorable equilibrium of ATPS reaction to the site of the products. Indeed, for enzymatic synthesis of APS the ATPS is coupled to pyrophosphatase to increase yields of the product [32]. Even more intriguing is a fusion protein of ATPS found in *Heterocapsa triquetra*. Here, ATPS is fused to APR. This fusion most probably ensures a rapid channelling of APS to the site of its reduction, increasing the rate of sulfite production. Existence of this fusion supports the hypothesis that the enzymes of sulfate assimilation may form a multi-enzyme complex as recent experiments in onion suggest [33].

As discussed before, plastidial ATPS isoforms from streptophytes resolved in a clade that contained only eukaryotes (Figure 3). Most likely the eukaryotic, host-encoded isoform was targeted to the plastid after endosymbiosis and the gene inherited from the cyanobacterial endosymbiont was lost. The enzymes found in chlorophytes, however, are not of the same origin as those found in plants. Instead, the chlorophytes encoded ATPSs are robustly related to isoforms found in algae with secondary plastids and to certain cyanobacteria.

### Evidence for a eukaryote to cyanobacteria lateral gene transfer

Association of plant and algal genes with cyanobacteria usually indicates a cyanobacterial endosymbiont (plastidial) origin, however, in the case of ATPS, this is unlikely for several reasons. Firstly, since primary plastids were likely acquired only once [34-36] this cyanobacterial enzyme would have been lost twice, independently, in red algae and in streptophytes, while chlorophytes must have lost the host isoform after the divergence of streptophytes. Secondly, the cyanobacteria in this clade do not branch at the base but are strongly supported as sisters to chlorophytes to the exclusion of other algae. Only two genera of marine cyanobacteria are represented in this clade: *Prochlorococcus *and *Synechococcus*, the other cyanobacteria being found elsewhere in the tree. Intriguingly, the same two genera were found to have obtained a fructose 1,6 bisphosphate aldolase from a red alga [37]. Thirdly, the clade also contains chromist algal species (haptophytes and stramenopiles) and dinoflagellates. These latter groups contain secondary plastids of red-algal origin [34,38,39] but the red algae are not present in this clade, meaning that inheritance through their plastids is unlikely.

The final piece of evidence against a cyanobacterial origin of the ATPS found in chlorophyte plastids is the sisterhood to the fungal and α-proteobacterial clade discussed above. It is not possible to state conclusively that the isoforms in the eukaryotes found in these clades are of prokaryotic origin. The tree, therefore, strongly suggests a lateral gene transfer of ATPS to these cyanobacteria, rather than cyanobacterial origin of this enzyme in plastids. However, if this is true, then the origin of ATPS in chlorophytes and algae with secondary plastids in unclear.

### Isoform retargeting of ATPS

In the red alga *Cyanidioschyzon merolae*, it appears that the different ATPS isoforms were subjected to a cellular targeting reversal. Two isoforms of ATPS were identified in the complete genome of this species. The first is quite distinct from that found in other lineages and related only to those encoded in other rhodophytes (Figure 3). The origin of this cytosolic ATPS in red algae remains unsolved, although it is perhaps most likely a derived paralogue of the second isoform, which resolved within a clade containing only eukaryotes. Intriguingly, although clustering with metazoans, this isoform encodes an N terminal sequence with all the features of a red-lineage transit peptide including a full 'FVAP' motif [24,40]. A similar phenomenon was observed in the diatom *T. pseudonana*, which also encodes two isoforms of ATPS. According to the currently predicted gene models, which manual examination could not fault, the first is fused at the N terminus to APS kinase and encodes an N terminal signal peptide, followed by a region with several features of a diatom transit peptide [41,42]. The second *T. pseudonana *isoform contains no extraneous N-terminal sequence by comparison to bacterial isoforms and is thus predicted to be cytosolic. The fused protein clusters, with good support, with aplastidic stramenopile relatives in the clade likely of eukaryotic origin (discussed above). This presumed cytosolic isoform is specifically related (100 maximum likelihood bootstraps) to another diatom (*P. tricornutum*) sequence, which does encode plastid-targeting pre-sequences and clusters with other plastid-targeted isoforms from a variety of algae, suggesting the targeting of the plastid and cytosolic isoforms were reversed in *T. pseudonana*.

This reversal of location of plastid and cytosolic isoforms must have occurred independently in *C. merole *and *T. pseudonana *and is highly unusual.

### Isoforms of plastid-targeted APR that do not bind an FeS cofactor are of PAPR origin

The enzymes catalyzing reduction of activated sulfate to sulfite, APR and PAPR are relatively similar sharing approx. 20% amino acid identity and a common active center represented by the amino acid sequence K/R E C G L/I H [10]. Initially, sulfate reduction was proposed to be PAPS dependent as the first organisms in which the pathway has been resolved were PAPR containing *Escherichia coli*, *Salmonella typhimurium *and *Saccharomyces cerevisiae *[10]. APS dependent assimilation of sulfate was, however, discovered in algae and plants [43] and thus it was believed that presence of plastids is the distinction between APS and PAPS pathways [10]. However, recently APRs were identified in a wide range of bacteria and the APS dependent reduction seems to be prevalent. While all fungi analyzed to date reduce PAPS, in prokaryotes the enzyme is limited to some groups of γ-proteobacteria, and cyanobacteria. The major difference between the two enzymes seemed to be the presence of [Fe_4_S_4_] cluster ligated by two invariant Cys pairs in all known APRs [6]. However, a novel isoform of the enzyme from the moss *Physcomitrella patens*, lacking the cluster, has been shown to reduce APS rather than PAPS [8]. This isoform was named APR-B to distinguish it from the majority of isoforms (denoted APR), which do require the presence of [Fe_4_S_4_]. It was identified in several other lower plant genera, such as *Selaginella *and *Marchantia *[44]. This demonstrates that whereas presence of the cluster is invariantly linked to use of APS as substrate, its absence is not a necessary determinant of PAPR. The recently sequenced genomes of several secondary algae and of the choanozoan *M. brevicollis *encode homologues of the APR-B isoform without the Cys pairs binding the FeS cluster. However, biochemical analysis revealed a high APR activity in the protein extracts of *T. pseudonana*, *E. huxleyi*, and *H. triquetra *[45] Kopriva S., unpublished). Position of these isoforms on the APR/PAPR phylogenetic tree was therefore of particular interest.

**Figure 4:**
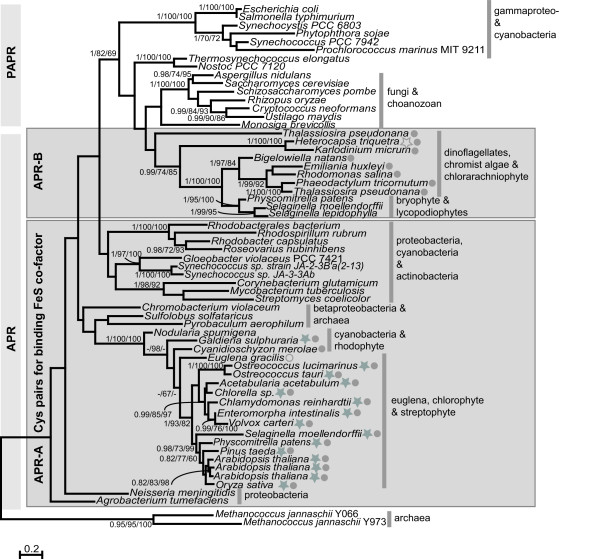
**Maximum likelihood phylogenetic tree of APR and PAPR**. The outgroup contains alignable sequences from archaea, annotated as APR/PAPR, but not proven experimentally. Numbers at nodes are (left to right) Bayesian posterior probabilities, maximum likelihood bootstraps and distance bootstraps. Support for nodes with less that 60 bootstraps by both methods are not show. Where tree topology differed or bootstraps by one method are less than 60, a dash is shown. A filled circle indicates the presence of a plastid targeting pre-sequence; an open circle indicates a mitochondrial targeting pre-sequence; a filled star indicates a *C *terminal thioredoxin moiety; an open star indicates fusion to ATPS. The shaded boxes contains isoforms that contain the sequence CCXXRR/K at positions 202–207 and GCXPCT at positions 293–98 (relative to *A. thaliana *AT4G04610) while those outside the shaded boxes contain the sequence Y/FXXXXK and GDXXXT/S at each position respectively. The bar indicates the number of amino acid changes per branch length.

Our analysis revealed that the PAPR found in fungi and *M. brevicollis *show specific relationship while that of *P. sojae *is found among prokaryotes, specifically cyanobacteria and γ-proteobacteria (Figure 4). This does not, however, give any support to a plastid origin for this gene since the red algae, to which the plastids found in other heterokonts are related, are found elsewhere in the tree. The *P. sojae *branch within the cyanobacteria is weakly supported (70 maximum likelihood bootstraps) although the clade, which also includes γ-proteobacteria is supported strongly (100 maximum likelihood bootstraps). The *P. sojae *enzyme is on a long branch and artifactual attraction to the cyanobacteria is also possible although tests by the artificial creation of other long-branches by the deletion of taxa (not shown) did not break the relationship indicating that lateral gene transfer is a possibility.

The APR of organisms with primary plastids can be found in the same clade in the expected order (rhodophytes at the base and chlorophytes and streptophytes as sister sub-clades). As stated above, aplastidic eukaryotes appear to reduce PAPS supporting that this APS reducing enzyme is likely of endosymbiont (plastid) origin. However, the origin of this plastid-targeted isoform is only supported by a single cyanobacterial sequence (*Nodularia*). Cyanobacteria have been found to reduce either PAPS or APS [46]. Interestingly, almost all cyanobacterial genomes sequenced to date encode a PAPR. On the other hand, biochemical analysis revealed APS reduction in many cyanobacteria such as *Symploca*, *Nodularia*, *Leptolyngbya*, or *Chroococcus *(Wiedemann G., Kopriva S., unpublished) but very little sequence data exists for these taxa and the amplification of the gene remains elusive. A fragment of a gene for APR from APS-reducing *Plectonema *has been cloned and sequenced and shown to branch at the base of the streptophyte APR clade in a previous, though restricted, analysis [6], however the sequence was too short for inclusion in this analysis. Since the *Nodularia *sequence is found at the base of the archaeplastidial clade, it is still possible that the APR in this lineage was obtained from their primary cyanobacterial endosymbiont.

The APR-B enzymes found in *P. patens *and the two *Selaginella *species branched outside of the well-supported clade that includes APR from plants. They were instead strongly related (100 maximum likelihood bootstraps) to chromist and chlorarachniophyte algae with dinoflagellates and an additional stramenopile isoform branching with good support (74 maximum likelihood bootstraps) at the base. This clade is sister to a clade containing PAPRs, the relationship of all being moderately well-supported (82 maximum likelihood bootstraps). Despite the lack of the Cys pairs for binding an FeS cluster, all of the enzymes within this clade are most likely to reduce APS rather than PAPS because, firstly, this has been demonstrated in *P. patens *and *S. lepidophylla *[8], secondly, as mentioned above, high APR activity has been measured in the chromist algae and lastly, the fusion of *H. triquetra *enzyme with ATPS indicates channeling of APS to its active centre. Still, detailed biochemical analysis of these proteins is needed to answer the question of substrate specificity unequivocally.

Two possibilities to explain this distribution exist. Firstly, the cyanobacterial ancestor of primary plastids contained APR, whereas the host that engulfed it possessed PAPR. The PAPS dependent enzyme changed its substrate specificity to APS and was then lost independently, a minimum of four times in rhodophytes, chlorophytes (after the branching of streptophytes), streptophytes (after the branching of bryophytes and lycopodiophytes leaving only APR in spermophytes) and in the chromalveolates/rhizarians, which comprise the rest of the clade. The second, more parsimonious explanation is a lateral gene transfer at the base of streptophytes from an alga with a secondary plastid, and the subsequent loss after the divergence of lycopodiophytes. This transfer would have had to happen after the change of substrate specificity. Better sampling of early diverging streptophytes might help to distinguish these possibilities, however, the strongly supported position of the mosses between the dinoflagellates and chromist (and chlorarachniophyte) algae, most strongly supports the latter.

The isoform of APS-reductase found in the chlorarachniophyte by *B. natans *is specifically related to those from chromist algae. The close association of chlorachniophytes and stramenopiles, both at the host level and by frequent lateral transfers between the two, has previously been noted [47-49].

### Sulfite Reductase

**Figure 5:**
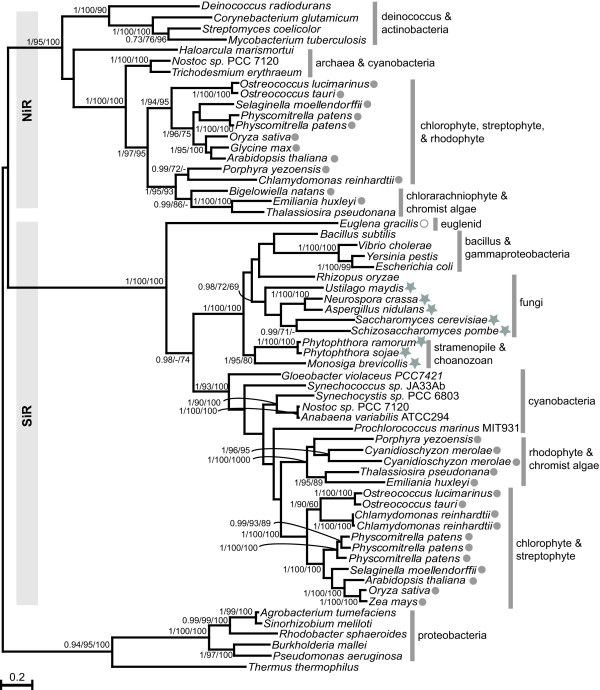
**Maximum likelihood phylogenetic tree of SiR and NiR**. The outgroup contains alignable sequences from proteobacteria that reduce both sulfite and nitrite. Numbers at nodes are (left to right) Bayesian posterior probabilities, maximum likelihood bootstraps and distance bootstraps. Support for nodes with less that 60 bootstraps by both methods are not show. Where tree topology differed or bootstraps by one method are less than 60, a dash is shown. A filled circle indicates the presence of a plastid targeting presequence; an open circle indicates a mitochondrial targeting presequence; a filled star indicates an *N *terminal pyruvate ferredoxin oxidoreductase moiety. The bar indicates the number of amino acid changes per branch length.

Sulfite and nitrite reductases are closely related proteins that are capable of reducing the reciprocal substrate, albeit at lower efficiency [1]. It is not always possible to conclude if an unknown sequence is a sulfite or nitrite reductase from primary sequence analyses such as BLAST. Therefore, to ensure the correct identification of new isoforms, such as that from *E. gracilis*, it was prudent to include both enzymes in the analysis. Since the subject of this manuscript is sulfate assimilation comments are limited to the SiR clade, which resolved with 100% bootstrap support (Figure 5).

All of the aplastidic eukaryotes which encode SiR showed a specific and well-supported relationship. This clade was sister to the CysI subunit of SiR from various proteobacteria. The SiR of cyanobacteria on the other hand showed closer relationship to plastid-containing eukaryotes. This division of prokaryotes and eukaryotes in Figure 5, rather than the separation of the two groups, suggests that all eukaryote SiRs are either of prokaryotic origin, inherited from the progenitors of the mitochondrion and/or plastid, or that lateral gene transfers have occurred. While the plastidial isoforms of SiR show strong relationships to cyanobacteria, the tree does not specifically support a mitochondrial origin for those found in aplastidic eukaryotes, although it is not ruled out.

The SiR encoded in *E. gracilis *showed no relationship to plastidial isoforms or to those from other eukaryotes, instead it branched at the base of the SiR clade. Although its position could be due to being derived, it could also indicate the only remaining eukaryotic isoform. Since no other sulfate assimilation genes were found among excavate sequences it is not possible to confirm whether this is the case. The tree supports neither a plastidial nor mitochondrial origin nor yet acquirement by lateral transfer. However, there is no evidence of a eukaryotic origin of either sulfite- or nitrite-reductases.

### Gene replacement from endosymbiont and retargeting of two enzymes in Euglena

The closest relatives of *Euglena *for which there is sequence data are the kinetoplasts. The complete genomes of *Trypanosoma *spp. and *Leishmania *spp., however, do not contain the enzymes for the reduction of sulfate. *Euglena *species differ from their closest relatives in that they contain a secondary plastid of green algal origin [50].

The ATPS encoded by *E. gracilis *is, along with other known enzymes of the pathway, preceded by a pre-sequence that suggests a mitochondrial location (Figure 2). Indeed, sulfate reduction activity in the mitochondria has been shown in this species [19,21]. The phylogenetic position within the algal-plastid (including chlorophyte) clade, makes a plastid origin (and therefore subsequent retargeting to mitochondria) of the enzyme most likely. However, as discussed above, the origin of this isoform in green algae is unclear. A plastid origin for the APR encoded by *E. gracilis *is also supported, with the enzyme being related to that found in the Archaeplastidal lineage, although the relationship to chlorophytes is not specifically supported. The origin of the third enzyme in the pathway, SiR, however, could not be determined, but very it is likely it is to be different from the ATPS and APR. Plastid localized sulfite reductase is ferredoxin dependent, whereas in *Euglena *sulfite reduction does not require ferredoxin and is thus most probably NADPH dependent [19]. The location of SiR in mitochondria suggests that the enzyme may be coupled to the unusual pyruvate:NADP^+ ^oxidoreductase, an ancient enzyme involved in oxidative decarboxylation of pyruvate [26]. This would explain not only the localization of SiR but also the rationale for *Euglena *not retaining and retargeting the protein inherited from its secondary plastid.

Although the full genomic sequence of *Euglena *is lacking, we can surmise that after the secondary endosymbiosis and subsequent relocation of genetic material to the host, the enzymes for ATPS and APR were re-targeted to the mitochondria, rather than to the compartment that they were inherited from and, likely, replaced the host-encoded genes. Because of lack of information on related host cells, it is unknown if the host reduced sulfur in the mitochondria prior to this event. Regardless, the location of the pathway in this cellular compartment raises some interesting biochemical questions. This localization of sulfate reducing centre ensures an effective provision of reduced sulfur to the cytosol and mitochondria, as demonstrated by the incorporation of the label from [^35^S] to free cysteine and proteins, respectively [21]. However, transport processes to import the reduced sulfur compounds to plastids are still necessary. This leads to the question does the mitochondrial location in Euglenids provide a more efficient ATP production and/or reducing capacity than it would in the plastid? And, if so, why? The answer may lie in understanding whether the mechanism of sulfate reduction in *Euglena *is the same as in other plastid containing eukaryotes. A detailed analysis of purified APR from *Euglena *revealed that the enzyme forms a reaction intermediate where sulfate from APS is covalently attached to a Cys residue of the protein, similar as in higher plant APR [51,52]. However, part of the sulfate is bound to a thiol-containing low molecular weight carrier, which does not seem to play any role in plant APR reaction [51,52]. As *Euglena *possesses a thiosulfonate reductase [19] the bound-sulfite pathway of sulfate assimilation, as previously proposed [53] may still exist in this species. The availability of the molecular tools will allow these intriguing questions to be addressed in the future.

## Conclusion

The inheritance of ATPS is complex, with multiple origins in the lineages that comprise the opisthokonts, different isoforms in chlorophytes and streptophytes, independent gene fusions in two species with other enzymes of the pathway, evidence of a eukaryote to prokaryote lateral gene transfer and two reversals of cellular location of host- and endosymbiont-originating enzymes. The evolution of APR and the related PAPR appears equally notable with evidence of lateral gene transfers and a recently described novel isoforms of APR from Bryophytes being more related to PAPRs found in fungi and bacteria than to the higher plant APRs indicating a change of substrate specificity during evolution.

We also found that the APTS and APR active in the mitochondria of *Euglena *were inherited from the secondary green algal plastid. We therefore confirmed the plastid localization of APR in chloroplasts of *C. reinhardtii *suggesting that in *Euglena *the proteins were retargeted to the mitochondria after the secondary endosymbiosis. *Euglena *SiR, on the other hand, is less related to plastidial isoforms and may be of host origin. Since all other eukaryotic lineages appear to encode isoforms inherited from either the mitochondrial or plastid endosymbiont origins, it might be the only known isoform of host origin. The many distinct isoforms of the three enzymes identified here represent an excellent resource for future studies

## Methods

### Immunolocalization of APR and SiR in *Chlamydomonas*

Fixed *C. reinhardtii *cells embedded in LR-Gold medium were kindly received from Peter Beech, Deakin University, Australia. Briefly, these cells were fixed with 0.75% glutaraldehyde in 10 mM Hepes (pH 7.4), ethanol dehydrated and embedded in LR-Gold resin. After sectioning, cells were blocked with 0.1% BSA and 0.001% Tween 40 in 1× PBS for one hour. Antibodies raised in rabbit against recombinant *Arabidopsis thaliana *APR2 or SIR [54], which identified single bands in *C. reinhardtii *protein extracts by Western blotting (Additional File 1), were applied for two hours at 25°C at 1:4000 (APR) and 1:2000 (SiR) dilutions. Following four five-minute washes in blocking buffer, secondary antibodies conjugated to 18 nm gold particles were applied overnight at 4°C, washed as before, and then stained with uranyl acetate for 60 seconds. As a control, sections were blocked and incubated with secondary antibodies only. Sections were viewed with a Philips CM120 BioTWIN transmission electron microscope. Particles of label were counted from three 1 μm^2 ^areas from three area of the cell (pyrenoid and starch sheath, chloroplast excluding pyrenoid and starch sheath, outside of chloroplast) from three cells, for each antibody (APR and SiR) and negative control (secondary antibody only). The results are given in Additional File 2.

### Sequence identification

Genomes were searched by systematic BLAST comparisons to proteins from *A. thaliana *and *Saccharomyces cerevisiae*. N-terminal targeting sequences given in Table 1 were predicted using iPSORT [55] and TargetP [56]. Sequences from *Homo sapiens, Dictyostelium discodium, Emiliania huxleyi, S. cerevisiae, Aspergillus nidulans, Oryza sativa *and *Selaginella moellendorffii *were obtained from NCBI sequence repository. Sequences from *Phytophthora sojae, Thalassiosira pseudonana, C. reinhardtii, Ostreococcus tauri, Osterococcus lucimarinus *and *Physcomitrella patens *were obtained from the Joint Genome Institute data repository [57]. Sequences from *Cyanidioschyzon merolae *were obtained from the *C. merolae *genome project [58]. Sequences from *Toxoplasma gondii *were obtained from ToxoDB [59]. Sequences from other, incomplete genomes used in phylogenetic analysis were obtained by BLAST comparisons to the protein and EST databases at NCBI. Sequences from *E. gracilis *and *Bigelowiella natans *were obtained from the tbEST database [60]. An incomplete expressed sequence tag clone of APR from *E. gracilis *was sequenced to completion and deposited in GenBank under accession number EU131629.

### Alignments and phylogenetic analysis

Protein alignments were made using Clustal X [61] and manually edited in MacClade (Sinauer, Mass. USA). All ambiguous sites of the alignments were removed from the dataset for phylogenetic analyses, otherwise the complete proteins were used for analysis. The final matrix size for APR and PAPR was 63 × 168; for ATPS it was 64 × 300; for SiR and NiR 62 × 387. The alignment data are provided in Additional File 3.

Protein maximum-likelihood analyses were performed using PhyML [62] with the WAG model of amino acids substitution, proportion of variable rates estimated from the data, and nine categories of substitution rates. 100 bootstrap trees were calculated with PhyML, also with nine rates categories. Bayesian trees were estimated by MrBayes version 3.1c using the WAG model with among-site rate variation modelled by a four discrete rates approximation of a gamma distribution. Four independent chains with random starting trees were run for 1 000 000 generations, samples were taken every five hundredth generation and the first 200 sampled trees were discarded as burn-in.

For distance analyses, gamma-corrected distances were calculated by PUZZLE using the WAG substitution matrix with eight variable rate categories and invariable sites. Trees were inferred by weighted neighbor-joining using WEIGHBOR and FITCH. Bootstrap resampling was performed using PUZZLEBOOT with rates and frequencies estimated using PUZZLE. Both distance trees gave the same topologies at supported nodes as maximum likelihood and Bayesian methods.

## Abbreviations

APS – adenosine 5'phosphosulfate; PAPS – 3'phosphoadenosine 5'phosphosulfate; APR – adenosine 5'phosphosulfate reductase; PAPR – 3'phosphoadenosine 5'phosphosulfate reductase; ATPS – ATP sulfurylase; SIR – sulfite reductase; OAS – *O*-acetylserine; OASTL – *O*-acetyl(homo)serine-(thiol)lyase

## Authors' contributions

NJP and SK conceived the study. DJD obtained and provided the sequence data for *Euglena gracilis*. NJP made sequence alignments and phylogenetic analyses and localization studies in *Chlamydomonas reinhardtii*. All authors were involved in interpreting the results and contributed to the concept of this manuscript. NJP and SK drafted the manuscript. All authors commented on drafts and approved the final manuscript.

## Supplementary Material

Additional file 1 Immunoblots of protein extracts of (A) *C. reinhardtii *and (B) *A. thaliana *with antibodies to APR and SiR. Click here for file

Additional file 2 Average and standard deviation (in parentheses) of numbers of immunogold label in TEM micrographs of of C. reinhardtii. Click here for file

Additional file 3 Alignment files (interleaved) with all ambiguously aligned characters removed of (A) ATPS, (B) APR and PAPR and (C) SiR and NiR. Click here for file
