# Supplementary figures and images for: Sulfate assimilation in eukaryotes: fusions, relocations and lateral transfers
Source: BMC Evol Biol. 2008 Feb 4;8:39. doi: 10.1186/1471-2148-8-39 (PMC2275785; doi:10.1186/1471-2148-8-39)

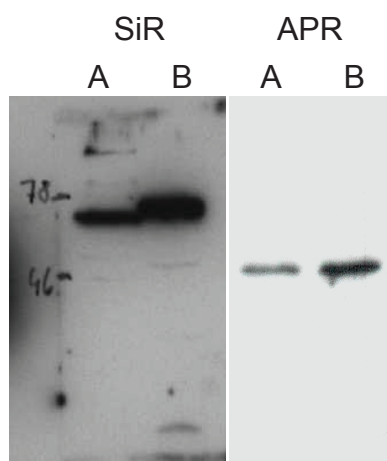

Supplement: Additional file 1 — Immunoblots of protein extracts of (A) C. reinhardtii and (B) A. thaliana with antibodies to APR and SiR. [file 1471-2148-8-39-S1.pdf]
