# Supplementary material for: Sulfate assimilation in eukaryotes: fusions, relocations and lateral transfers
Source: BMC Evol Biol. 2008 Feb 4;8:39. doi: 10.1186/1471-2148-8-39 (PMC2275785; doi:10.1186/1471-2148-8-39)
Supplement: Additional file 2 — Average and standard deviation (in parentheses) of numbers of immunogold label in TEM micrographs of of C. reinhardtii. [file 1471-2148-8-39-S2.pdf]

Additional File 2

Average and standard deviation (in parentheses) of gold label particles per one μm^2 of three cell areas (A= pyrenoid and starch sheath, B chloroplast excluding pyrenoid and starch sheath C= cell, non-chloroplast).

Three 1μm^2 areas of each type were counted from three cells for each repetition (experiment 1 and 2) and for each antibody (APR and SiR) and negative control (secondary antibody only).

		APR	SiR	Control
Experiment 1	A	23.7 (6.1)	19.9 (5.1)	0
	B	4.3 (1.7)	5.6 (2.8)	0
	C	0.9 (1.1)	1.4 (1.7)	0.08 (0.2)
Experiment 2	A	22.7 (6.1)	21.7 (7.0)	0
	B	9.2 (2.2)	6.7 (3.4)	0
	C	0.58 (1.2)	0.16 (0.3)	0
